# AAV9-mediated Rbm24 overexpression induces fibrosis in the mouse heart

**DOI:** 10.1038/s41598-018-29552-x

**Published:** 2018-08-03

**Authors:** Maarten M. G. van den Hoogenhof, Ingeborg van der Made, Nina E. de Groot, Amin Damanafshan, Shirley C. M. van Amersfoorth, Lorena Zentilin, Mauro Giacca, Yigal M. Pinto, Esther E. Creemers

**Affiliations:** 1 Department of Experimental Cardiology, Academic Medical Center (AMC), Amsterdam, The Netherlands; 2 International Centre for Genetic Engineering and Biotechnology, Trieste, Italy

## Abstract

The RNA-binding protein Rbm24 has recently been identified as a pivotal splicing factor in the developing heart. Loss of Rbm24 in mice disrupts cardiac development by governing a large number of muscle-specific splicing events. Since Rbm24 knockout mice are embryonically lethal, the role of Rbm24 in the adult heart remained unexplored. Here, we used adeno-associated viruses (AAV9) to investigate the effect of increased Rbm24 levels in adult mouse heart. Using high-resolution microarrays, we found 893 differentially expressed genes and 1102 differential splicing events in 714 genes in hearts overexpressing Rbm24. We found splicing differences in cardiac genes, such as PDZ and Lim domain 5, Phospholamban, and Titin, but did not find splicing differences in previously identified embryonic splicing targets of Rbm24, such as skNAC, αNAC, and Coro6. Gene ontology enrichment analysis demonstrated increased expression of extracellular matrix (ECM)-related and immune response genes. Moreover, we found increased expression of Tgfβ-signaling genes, suggesting enhanced Tgfβ-signaling in these hearts. Ultimately, this increased activation of cardiac fibroblasts, as evidenced by robust expression of Periostin in the heart, and induced extensive cardiac fibrosis. These results indicate that Rbm24 may function as a regulator of cardiac fibrosis, potentially through the regulation of TgfβR1 and TgfβR2 expression.

## Introduction

Alternative splicing, a process to generate multiple mRNA transcripts from a single gene, underlies many developmental processes and can contribute to disease progression and severity in the heart^1,2^. Several pivotal splicing factors, such as RNA-binding motif protein 20 (RBM20), RNA-binding motif protein 24 (RBM24) and Splicing factor 3B subunit 1 (SF3B1), have been identified in the heart, and we are only just beginning to understand the function of specific protein isoforms, induced by these splicing factors, for cardiac physiology^3–5^. For instance, mutations in RBM20 lead to an early onset dilated cardiomyopathy through missplicing of multiple cardiac genes such as Titin, CamkIIδ, and RyR2^3^. SF3B1 is an HIF-1α driven splicing factor which is required for proper cardiac metabolism^4^. Interestingly, cardiac-specific loss of SF3B1 protects against pathological hypertrophy and contractile dysfunction, due to splicing regulation of ketohexokinase, a key metabolic enzyme^4^. The RNA-binding protein Rbm24 is a critical regulator of cardiac lineage differentiation of human embryonic stem cells and heart development. It has recently been shown that Rbm24 is upregulated during cardiac differentiation of human embryonic stem cell derived cardiomyocytes^6^, where it regulates alternative splicing of pluripotency and sarcomeric genes during differentiation^7^. Specifically, Rbm24 overexpression promotes, whereas Rbm24 knockdown inhibits cardiac lineage differentiation in human embryonic stem cells, by regulating over 200 alternative splicing events^7^. Along the same lines, knockdown of Rbm24 in the developing zebrafish heart results in compromised cardiac contractility, attributed to impaired sarcomere formation and decreased expression of sarcomeric genes^8^. Similarly, targeted disruption of Rbm24 in mice leads to embryonic lethality, due to cardiac malformations and impaired sarcomerogenesis^5^. In the developing mouse heart, Rbm24 regulates at least 68 alternative splicing events, mostly by promoting muscle-specific exon inclusion. Several of these Rbm24-mediated splicing events, e.g. Naca, Fxr1, or Abcc9, have been reported to underlie cardiac development, sarcomere formation and cardiomyopathies^5^. However, apart from regulating mRNA splicing, it is known that Rbm24 can stabilize mRNA targets, such as p21 and myogenin^9,10^. For myogenin, it has for example been shown that Rbm24 increases the half-life of the myogenin mRNA transcript by binding to its 3′UTR, and thereby promoting myogenic differentiation in C2C12 cells. In line with its multiple functions, Rbm24 is localized both in the nucleus, where it can influence alternative splicing, and the in the cytoplasm, where it can stabilize mRNA targets^11^. Overall, Rbm24 is well established as a pivotal RNA-binding protein and splicing factor in cardiac and myogenic differentiation and in the developing heart.

The role of Rbm24 in the postnatal and adult heart, however, is yet unknown. We have recently shown that Rbm38, a closely related family member of Rbm24, is dispensable for normal cardiac function, both at baseline and after pressure overload-induced cardiac remodeling^12^. Rbm24 and Rbm38 share 68% of sequence identity, which suggests they could be genetically redundant^12^. We hypothesized that, since Rbm38 is dispensable for proper cardiac structure and function, Rbm24 might be more important in the adult heart. Therefore, we used adeno-associated virus serotype 9 (AAV9)-mediated overexpression of Rbm24, to examine its role in the early postnatal and adult mouse heart. We found that overexpression of Rbm24 increases cardiac fibrosis. We suggest that Rbm24 overexpression in the mouse heart increases the expression of Tgfβ- and extracellular matrix (ECM)-related genes, such as TgfβR1 and TgfβR2, and thereby activates collagen synthesis.

## Materials and Methods

### AAV generation

A flag-tagged (N-terminal) open reading frame of mouse Rbm24 (ENSMUST00000037923;NCBIm37) was cloned into the pZac2.1 vector (under control of a CMV-promotor) and was subsequently used for AAV generation. AAVs were generated by the AAV Vector Unit at ICGEB Trieste (http://www.icgeb.org/avu-core-facility.html) following a protocol as previously described^13^. AAVs encoding GFP were used as control.

### Mouse injections

Wildtype mice (C57/Bl6) were injected intraperitoneally with 2 × 10^12^ (low dose) or 4 × 10^12^ (high dose) viral genomes (vg) at 1 week of age, and sacrificed 2 weeks, 4 weeks, or 8 weeks after injection, after which heart and liver were harvested. Number of animals used per group: low dose 2 weeks: 4 AAV9-GFP and 4 AAV9-Rbm24, high dose 2 weeks: 4 AAV9-GFP and 4 AAV9-Rbm24, low dose 8 weeks: 3 AAV9-GFP and 5 AAV9-Rbm24, high dose 4 weeks: 6 AAV9-GFP and 6 AAV9-Rbm24. All animal studies were approved by the Institutional Animal Care and Use Committee of the University of Amsterdam, and in accordance with the guidelines of this institution and the Directive 2010/63/EU of the European Parliament.

### RNA isolation and (q)RT-PCR

RNA was isolated using TRIreagent (Sigma-Aldrich) using the manufacturer’s protocol. After DNAse (Invitrogen) treatment of 1 µg RNA, cDNA was generated with SuperScript II (Invitrogen). RT-PCRs were performed with Hot Fire Taq polymerase (Solis Biodyne) using standard protocols. qPCR was done using SYBR Green (Roche) on a LightCycler 480 II (Roche) and analysis was done using LinRegPCR software^14^. Primer sequences can be found in Supplemental Table 1.

### Protein isolation and Western blotting

Protein was isolated from heart tissue (right ventricle) by grinding the tissue in RIPA buffer (50 mM Tris-HCl, 150 mM NaCl, 1% NP-40, 0.2% sodium deoxycholate, 0.1%SDS, 1 mM Na3VO4, 1 mM PMSF) supplemented with protease inhibitor cocktail (Roche) with repeated freeze-thaw cycles. Protein lysates were cleared by centrifugation (14000 g for 15 min at 4 °C). Protein concentration was measured using the BCA protein assay (Pierce). Proteins were separated by SDS-PAGE and transferred to PVDF membranes (Bio-Rad). Membranes were blocked for 1 hr at RT, and overnight incubated with primary antibodies at 4 °C. The next day, membranes were washed with TBS-T (3 × 5 min) and incubated with a HRP-conjugated secondary antibody for 1 hr at RT. Western blots were developed using ECL prime western blotting reagent (Amersham Biosciences) and visualized using an ImageQuant LAS4000 (GE Healthcare Europe). Antibodies can be found in Supplemental Table 1.

### Histological analysis

Hearts were fixed overnight in 4% paraformaldehyde, transferred to 70% ethanol, dehydrated, and embedded using standard techniques. Sections of 5 µm were stained with Hematoxylin and Eosin for gross morphology and Picrosirius Red for fibrosis. Per section, 5 pictures were taken from the LV using a light microscope (20x magnification). Fibrosis quantification was done using an in-house macro in ImagePro 6.2^12^. Perivascular fibrosis was manually omitted from the pictures. Number of animals used per group: low dose 2 weeks: 4 AAV9-GFP and 3 AAV9-Rbm24, high dose 2 weeks: 3 AAV9-GFP and 4 AAV9-Rbm24, low dose 8 weeks: 3 AAV9-GFP and 5 AAV9-Rbm24, high dose 4 weeks: 6 AAV9-GFP and 4 AAV9-Rbm24.

### Immunohistochemistry

Sections of 5 µm were deparaffinized and rehydrated in a series of ethanol. Antigens were retrieved by boiling sections for 5 min in antigen unmasking solution (H3300, Vector) in a pressure-cooker. Permeabilization was done by incubating sections in PBS-0.1% Triton X-100 for 15 min at RT. Sections were then blocked in 4% normal goat serum (NGS) in PBS for 1 hr at RT, and incubated with primary antibodies in 4% NGS in PBS overnight at 4 °C. Alexa Fluor 488 and Alexa Fluor 647 conjugated antibodies (Invitrogen) were used as secondary antibodies, and nuclei were visualized using DAPI (Molecular Probes). Pictures were taken on a Leica SP8 confocal microscope (Leica Microsystems). Antibodies can be found in Supplemental Table 1. Percentage of AAV9-flag-Rbm24 infected cardiomyocytes was calculated manually using confocal microscopy by counting the flag- and α-actinin positive cardiomyocytes. Between 350 and 800 cells were counted per heart.

### Transcriptome analysis

RNA from 3 AAV9-GFP and 3 AAV9-Rbm24 injected mouse hearts (high dose, 2 weeks after injection) was used for micro-array analysis. RNA quality was measured using the Agilent Bioanalyzer (all RIN values > 8.5). Gene expression and alternative splicing was examined using an Affymetrix Mouse Transcriptome Array 1.0. Gene expression and alternative splicing analysis was performed using Expression Console Software and Transcriptome Analysis Console Software from Affymetrix. Genes with a fold chance of at least 1.5 and an ANOVA p-value < 0.05 were used for gene ontology enrichment. The following cut-offs were used for alternative splicing analysis: probe detected in all samples, ANOVA p-value < 0.01, splicing index > 2, and only coding or complex genes were analyzed. Gene ontology enrichment analysis was done using the online gene ontology enrichment analysis tool Panther^15^.

### Statistical analysis

Data are presented as mean ± sem, and Mann-Whitney U-test was used to test for statistical significance. A p-value < 0.05 was considered significant.

### Data availability

The datasets generated during and/or analyzed during the current study are available from the corresponding author on reasonable request. Micro-array data has been deposited at Geo Datasets under accession number GSE110991.

## Results

### AAV9-mediated overexpression of Rbm24 in the mouse heart

**Figure 1:**
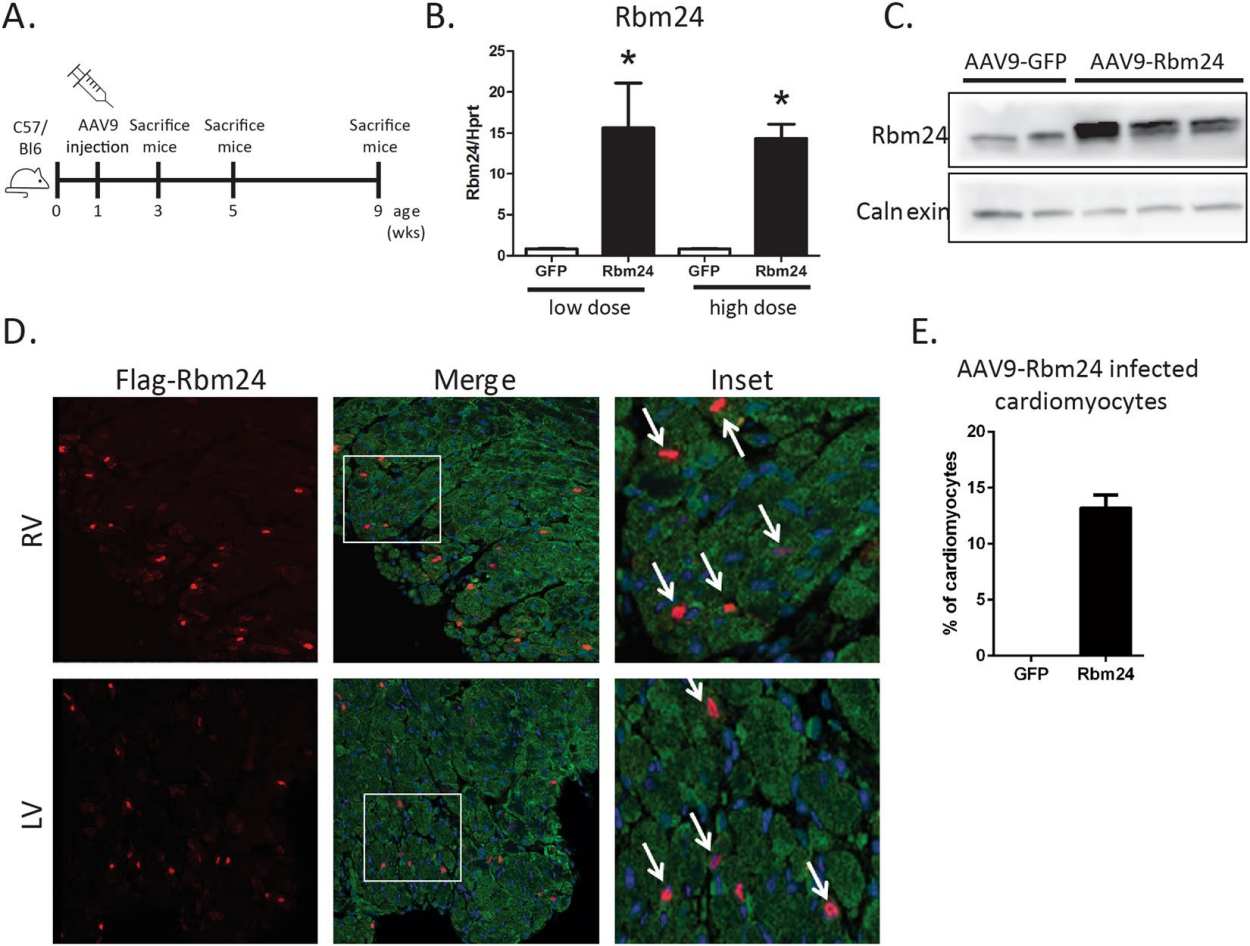
AAV9 injections in wildtype C57/Bl6 mice. (**A**) Experimental set-up. (**B**) qPCR analysis of Rbm24 in hearts of AAV9 injected mice, 2 weeks after injection (n = 3–4 per group). (**C**) Western blot of Rbm24 in hearts of AAV9 injected mice, 2 weeks after injection. (**D**) Immunohistochemistry of AAV9-induced Rbm24 with an anti-flag antibody (red). Sections were counterstained for α-actinin (green). Nuclei were stained with DAPI (blue). Inset: AAV9-infected cells were α-actinin positive, indicating that AAV9 infected cells are cardiomyocytes (white arrows). Sections were derived from hearts of AAV9 injected mice 2 weeks after injection. Magnification 40x. (**E**) Percentage of Flag-Rbm24 positive cardiomyocytes in the hearts of AAV9-infected mice.

**Figure 2:**
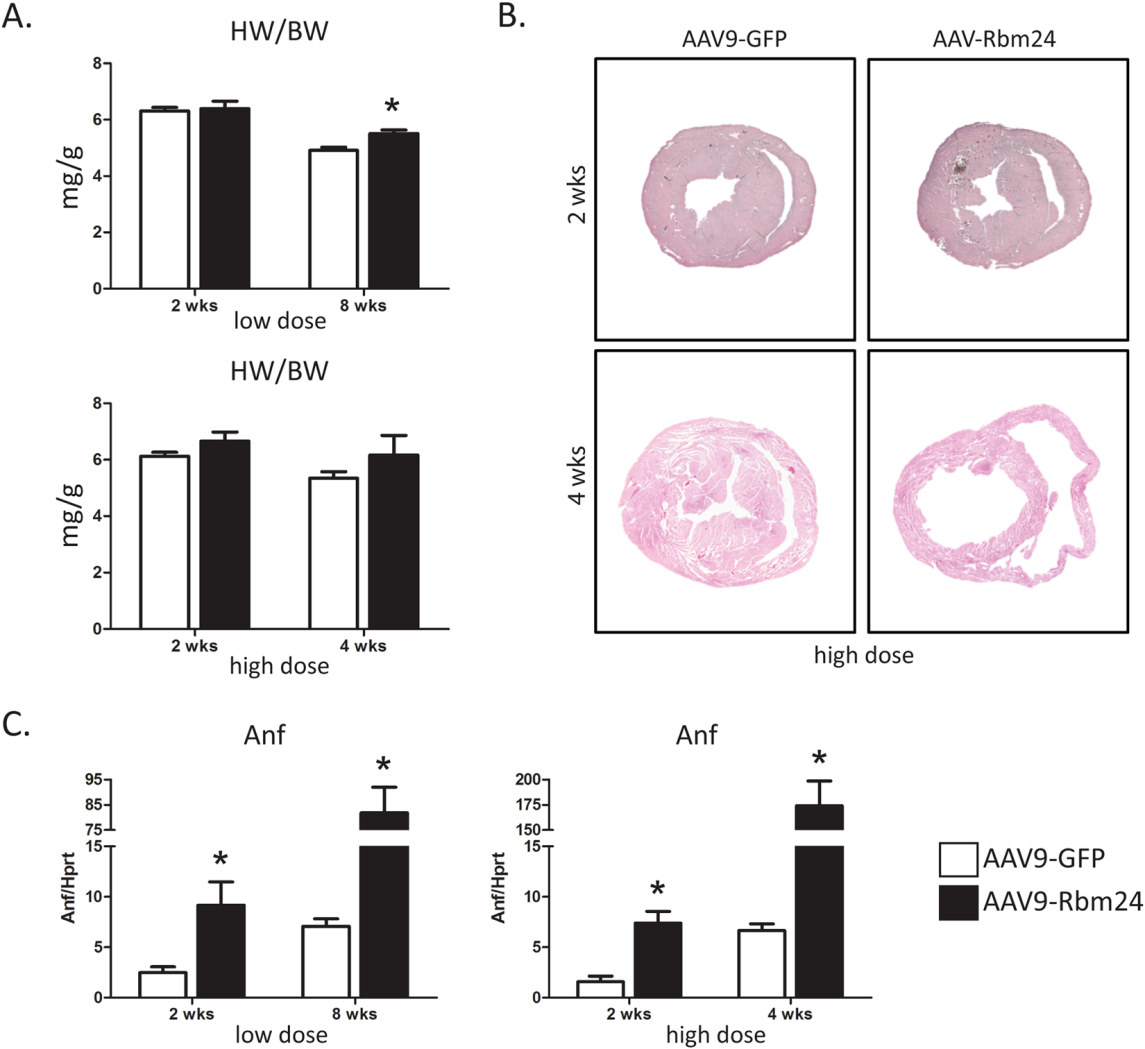
Cardiac phenotype of AAV9 injected mice. (**A**) Heart weight/body weight ratios of AAV9 injected mice (n = 3–6 per group). (**B**) H&E staining of hearts of AAV9 (high dose) injected mice, 2 and 4 weeks after injection. (**C**) qPCR analysis of Anf (Nppa) in the hearts of AAV9 injected mice (n = 3–6 per group).

In order to investigate a potential role for Rbm24 in the postnatal and adult heart, we generated AAV9 viruses encoding a flag-tagged open reading frame of mouse Rbm24. It has previously been shown that the AAV9 serotype preferentially infects cardiomyocytes, where it induces strong expression of the packaged genes^16,17^. We injected one-week-old wildtype mice intraperitoneally with a single bolus injection (2 × 10^12^ vg (low dose) or 4 × 10^12^ vg (high dose)) of either AAV9-GFP or AAV9-flag-Rbm24, and analyzed 2 weeks, 4 weeks, or 8 weeks later (Fig. 1A). Both the low dose and the high dose resulted in an approximately ~15-fold upregulation of Rbm24 mRNA in the heart, and Western blotting using an Rbm24 antibody showed that Rbm24 protein was efficiently produced in the hearts of animals that were transduced with AAV9-Rbm24 (Fig. 1B,C). We next examined the cellular origin of AAV9-induced flag-Rbm24, by co-immunohistochemical stainings with antibodies raised against flag and α-actinin, and revealed recombinant Rbm24 expression in ~13% of cardiomyocytes throughout the whole heart (Fig. 1D,E). Mice injected with the low dose showed no obvious abnormalities or signs of heart failure, but injection of the high dose caused mortality in 3 out of 6 mice after 3 to 4 weeks. Therefore, surviving mice injected with the high dose were sacrificed 4 weeks after injection, and mice injected with the low dose were sacrificed 8 weeks after injection. Mice sacrificed after 4 weeks or 8 weeks still overexpressed Rbm24, even though Rbm24 expression was less increased after 8 weeks than after 2 or 4 weeks after injection (Supplemental Figs 1 and 2). Heart weight/body weight (HW/BW) ratios were not different at 2 weeks after injection of the low dose of AAV9-Rbm24, but were slightly increased at 8 weeks after injection (Fig. 2A). After injection of the high dose, HW/BW ratios showed a trend towards an increased HW/BW ratio, both at 2 and 4 weeks after injection (Fig. 2A). It must be noted, however, that after injection of the high dose only mice that survived the first 4 weeks were analyzed, meaning that these results are biased towards mice that were least affected. Gross cardiac morphology was not different between AAV9-GFP and AAV9-Rbm24 at 2 weeks after injection of the low or high dose, as shown by H&E stainings (Fig. 2B). However, injection of the high dose resulted in severe cardiac dilatation and wall thinning at 4 weeks after injection (Fig. 2B). Even though we did not observe overt cardiac changes at 2 weeks after injection, we did find the stress marker Anf (or Nppa) to be upregulated, which was even further increased at 4 weeks and 8 weeks after injection, in the hearts of AAV9-Rbm24 injected mice (Fig. 2C). In conclusion, we generated an AAV9 virus encoding flag-Rbm24, which adequately induced Rbm24 expression throughout the heart. Injection of the low dose of AAV9-Rbm24 did not result in an overt cardiac phenotype at 2 or 8 weeks after injection, but did increase the stress marker Anf. Injection of the high dose of AAV9-Rbm24 did not result in an overt phenotype at 2 weeks after injection, but caused severe cardiac dilatation and mortality after 4 weeks.

### Gene expression and alternative splicing in AAV9-Rbm24 hearts

**Figure 3:**
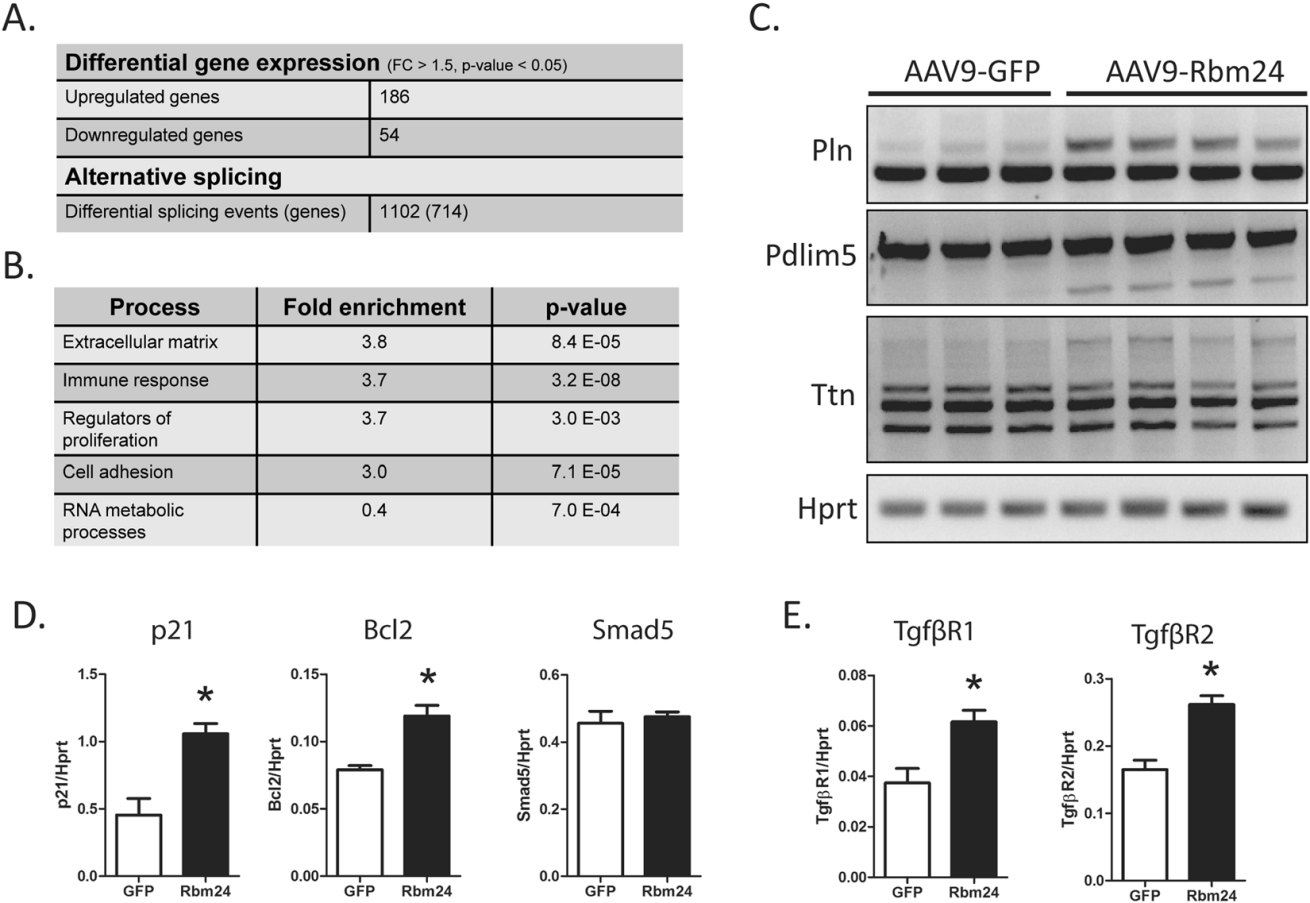
Transcriptome analysis of hearts of AAV9-Rbm24 injected mice. (**A**) Gene expression and alternative splicing differences the hearts of mice injected with the high dose of AAV9-Rbm24 compared to hearts of mice injected with AAV9-GFP, 2 weeks after injection. (**B**) Gene ontology enrichment analysis of differentially expressed genes in the hearts of mice injected with AAV9-Rbm24. (**C**) End-point RT-PCR analysis of Pln, Pdlim5, and Ttn. (**D**) qPCR analysis of previously identified mRNA targets p21, Bcl2, and Smad5 (n = 3–4 per group). (**E**) qPCR analysis of Tgfβ receptors 1 and 2 (n = 3–4 per group).

To interrogate alternative splicing events and gene expression changes in hearts of AAV9-Rbm24 injected mice, we isolated RNA from left ventricles and performed microarray analysis. We used the AffyMetrix Mouse Transcriptome Array 1.0, since this microarray platform contains approximately four probes per exon as well as probes that span exon-exon junctions, and roughly 40 probes per gene, and as such enables for high resolution gene expression and alternative splicing analysis. We first analyzed differential gene expression and found a total number of 646 genes to be at least 1.5-fold upregulated, of which 186 were protein coding. 247 genes were at least 1.5-fold downregulated, of which 54 were protein coding (Fig. 3A, Supplemental File 1). Gene ontology enrichment analysis using PANTHER revealed an enrichment of extracellular matrix (ECM) genes, immune response genes, and genes involved in cellular proliferation (Fig. 2B). Since Rbm24 is also known as a splicing factor, we further analyzed the micro-array results for differential splicing events. Using a stringent cut-off of at least a 2-fold difference of in- or exclusion of an exon, we identified 1102 differential splicing events in 714 genes (Fig. 3A, Supplemental File 1). We did not observe splicing differences in the known Rbm24-splicing targets skNAC, αNAC, and Coro6, which were recently identified in embryonic Rbm24 knockout hearts (Supplemental Fig. 3)^5^. This is, however, not surprising, as these splice isoforms are induced by Rbm24 in the developing heart, and remain to be expressed during adulthood. Increasing expression of Rbm24 postnatally does therefore likely not add to the induction of these splice isoforms, since the switch in splice isoform has already taken place. We noticed that some of the genes with the largest splicing differences (i.e. the highest splicing index) were pivotal cardiac genes, such as Pln, Pdlim5, and Ttn, that have all been implicated in cardiac disease. We could validate these splicing differences with RT-PCR in hearts with increased Rbm24 expression (Fig. 3C). Recent reports have shown that Rbm24 controls mRNA expression of multiple genes such as p21, Bcl2, and Smad5^8,10^. To investigate whether these previously identified targets were also regulated in our model, we analyzed the mRNA expression of these genes. We found that p21 and Bcl2, but not Smad5, were upregulated in the AAV9-Rbm24 hearts at 2 weeks after injection (Fig. 3D), which is in line what has been found previously^8,10^. In addition, the expression of p21 and Bcl2 was also increased at 4 or 8 weeks after AAV9-injection (Supplemental Figs 1 and 2).

### Rbm24 overexpression induces fibrosis

**Figure 4:**
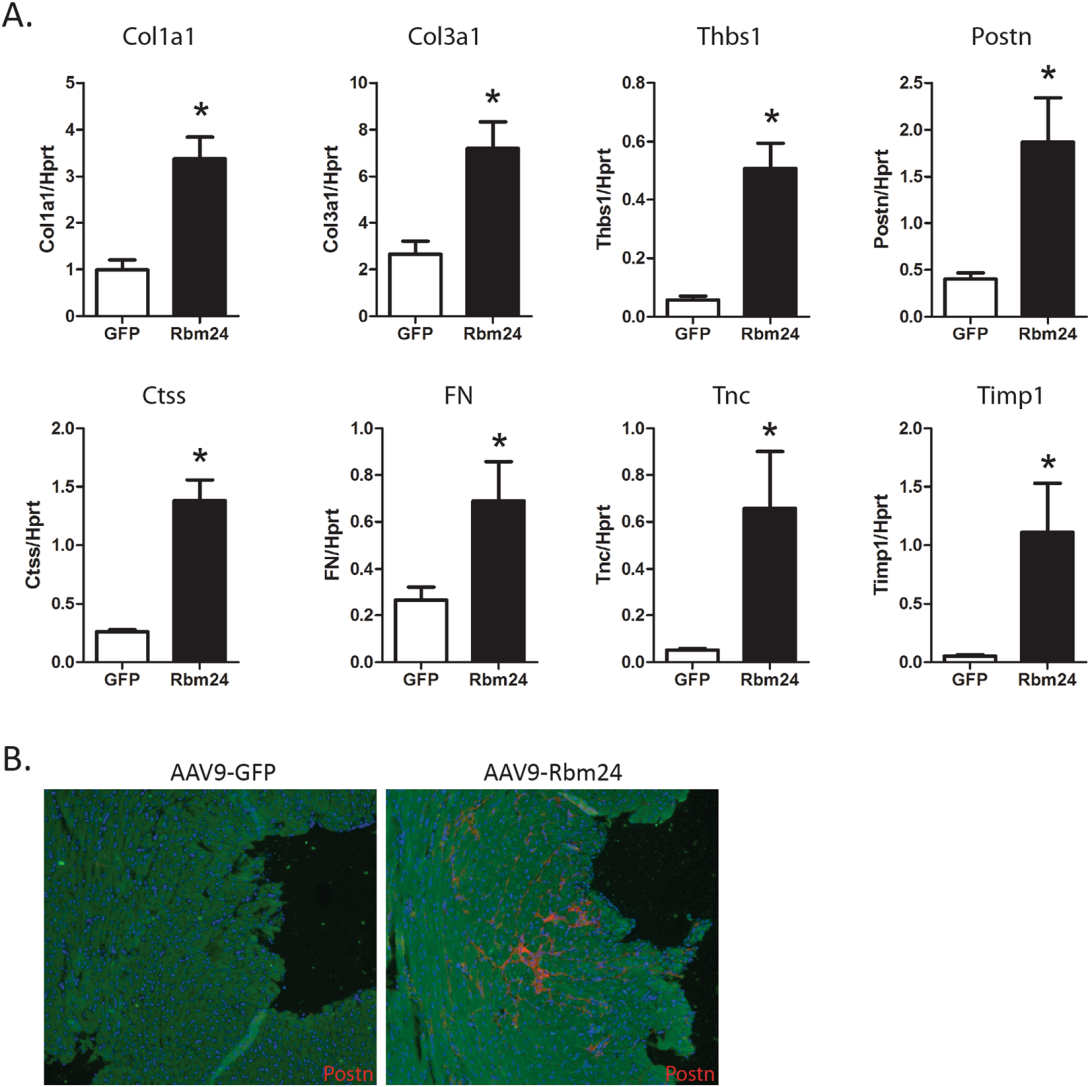
Expression of extracellular matrix genes. (**A**) qPCR analysis of ECM genes in the hearts of AAV9 injected mice, 2 weeks after injection (n = 3–4 per group). (**B**) Immunohistochemistry of Postn (red) in the hearts of AAV9 injected mice, 2 weeks after injection. Hearts were counterstained for α-actinin (green). Nuclei were stained with DAPI (blue). Magnification 40x.

**Figure 5:**
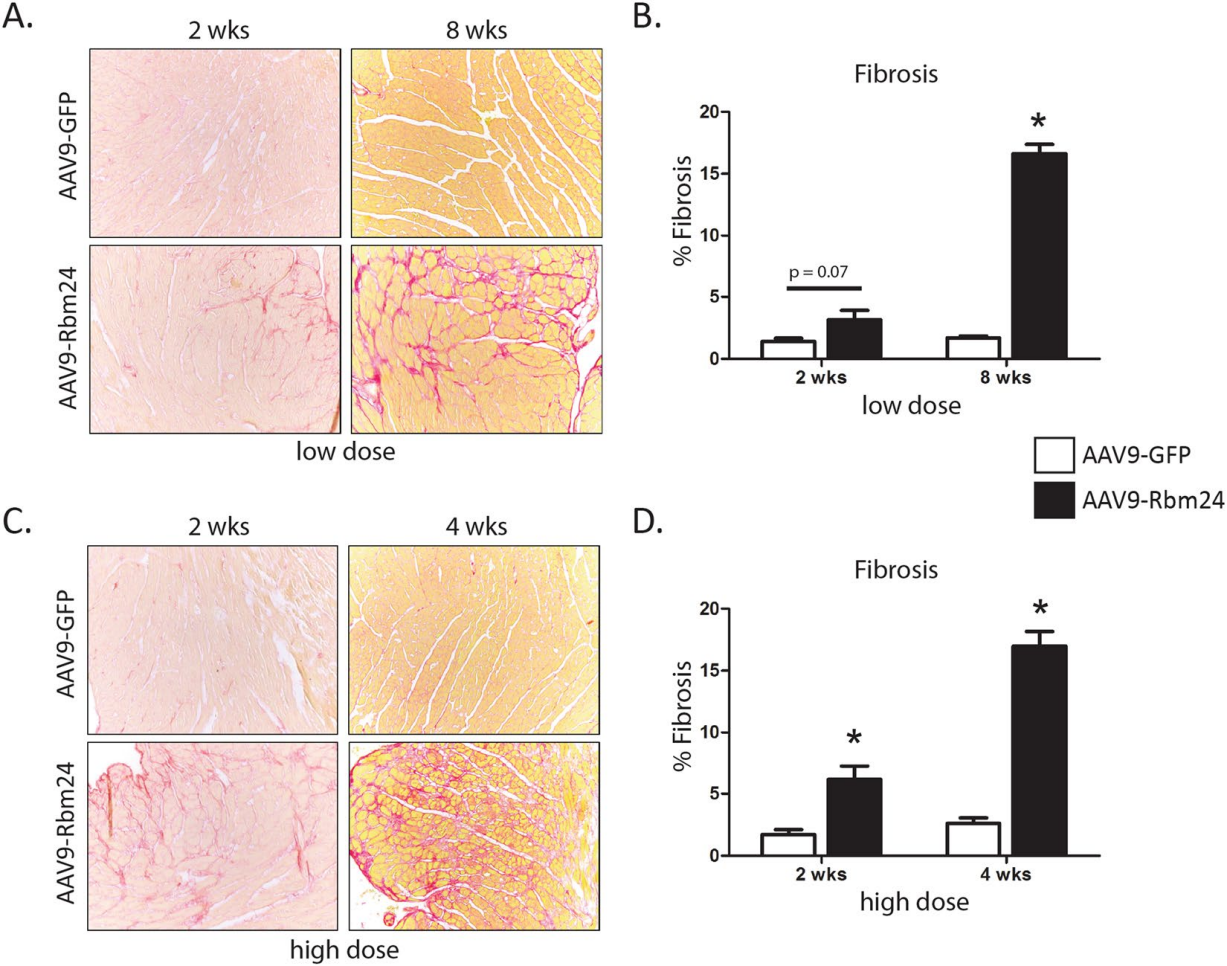
Rbm24 overexpression induces fibrosis. (**A**) Picrosirius Red staining of hearts of AAV9 (low dose) injected mice, 2 and 8 weeks after injection. (**B**) Quantification of Picrosirius Red staining of hearts of AAV9 (low dose) injected mice, 2 and 8 weeks after injection. (**C**) Picrosirius Red staining of hearts of AAV9 (high dose) injected mice, 2 and 4 after injection. (**B**) Quantification of Picrosirius Red staining of hearts of AAV9 (high dose) injected mice, 2 and 4 weeks after injection.

We noted that multiple genes from the Tgfβ pathway, such as Tgfβ2 and TgfβR2, were increased in Rbm24-overexpressing hearts (Supplemental File 1). We validated two of these genes using qPCR, and indeed found an increase in TgfβR1 and TgfβR2 expression, two weeks after AAV9-Rbm24 injections (Fig. 3E). After 4 and 8 weeks, the expression of TgfβR1 remained high, while the expression of TgfβR2 returned to control levels (Supplemental Figs 1 and 2). Increased expression of Tgfβ receptors could lead to enhanced Tgfβ signaling, which, in turn, could contribute to increased expression of ECM genes. Since we found ECM genes to be enriched in the AAV9-Rbm24 hearts in our microarrays, we next aimed to validate the expression of a set of ECM genes by qRT-PCR. Indeed, we confirmed increased expression of a wide range of ECM and fibrotic genes already at 2 weeks after injection, and these genes were even further upregulated 4 and 8 weeks after injection (Fig. 4A, Supplemental Figs 4 and 5). One of the upregulated genes, periostin (Postn), marks activated fibroblasts, which are crucial in the fibrotic response. Since Postn mRNA is upregulated in the AAV9-Rbm24 injected hearts, we performed co-immunohistochemistry with a Postn and α-actinin antibody, and found Postn protein expression markedly enhanced in the AAV9-Rbm24 injected hearts, indicating an active fibrotic response after Rbm24 overexpression (Fig. 4B). The increased expression of TgfβR1 and TgfβR2, and of ECM-related genes in the hearts of AAV9-Rbm24 injected mice point towards an activated fibrotic response. Therefore, we stained cardiac sections with Picrosirius Red, and found extensive fibrosis in the hearts of AAV9-Rbm24 injected mice, but not in the AAV9-GFP injected mice. Quantification of these Picrosirius Red stainings revealed a ~2-fold increase in collagen content 2 weeks after injecting the low dose and a ~10-fold increase in mice 8 weeks after injection of the low dose (Fig. 5A,B). Mice injected with the high dose of AAV9-Rbm24 showed a ~3-fold increase at 2 weeks after injection, and a ~7-fold increase at 4 weeks after injection (Fig. 5C,D). Overall, we show that increased expression of Rbm24 in the early postnatal and adult mouse heart increases cardiac fibrosis.

## Discussion

We investigated the role of the RNA-binding protein and splicing factor Rbm24 in the early postnatal and adult heart. We found that AAV9-mediated overexpression of Rbm24 in the mouse heart increases the expression of Tgfβ- and ECM-related genes. We observed increased activation of cardiac fibroblasts, as evidenced by robust expression of Postn in the heart, and extensive cardiac fibrosis in AAV9-Rbm24 injected mice. These results suggest that Rbm24 may function as a novel regulator of cardiac fibrosis, potentially through the regulation of TgfβR1 and TgfβR2 expression. It is intriguing that only injection of the high dose causes lethality, while the overexpression of Rbm24 is similar at 2 weeks after injection. It is not clear what underlies the lethality, but it may be possible that the high dose leads to higher Rbm24 levels at later time-points. Another possibility is that injection of the high dose of AAV9-RBM24 triggers an immune response to the AAV vector capsid or to the transgene product, leading to release of inflammatory cytokines, cell death, and cardiac dilation. However, the induction of the pro-fibrotic program seemed independent of the lethal cardiomyopathic phenotype, as this was consistent across dosages and time-points.

Extensive cardiac fibrosis directly hampers cardiac function, since the deposition of extracellular matrix stiffens the ventricular wall and reduces compliance^18,19^. In addition, extensive cardiac fibrosis is also pro-arrhythmic^18,19^. One of the major drivers of cardiac fibrosis is Tgfβ signaling, which can work via canonical (Smad-dependent) and non-canonical (Smad-independent) pathways^20^. In the canonical pathway, activation of TgfβR1 and TgfβR2 by Tgfβ instigates phosphorylation of Smad2 and Smad3, which subsequently translocate to the nucleus together with Smad4, to induce gene expression of Tgfβ-responsive genes such as Col1a1, Col3a1, and fibronectin^20,21^. In the non-canonical pathway, activated Tgfβ receptors directly activate Smad-independent signaling pathways, such as the MAPK cascade and Erk signaling^22^. Interestingly, both cardiomyocyte specific knockout of TgfβR2, and fibroblast specific knockout of TgfβR1 and TgfβR2, are protective against pressure-overload induced cardiac fibrosis, albeit through different mechanisms^23,24^. In the cardiomyocyte, TgfβR2 deficiency inhibits both canonical and non-canonical Tgfβ signaling, but non-canonical signaling through TAK1 is necessary for the protective effect against cardiac fibrosis^24^. In the cardiac fibroblast, on the other hand, canonical Tgfβ signaling through Smad2/3 underlies cardiac fibrosis^23^.

The observation that overexpression of Rbm24 in the mouse heart increases the expression of Tgfβ- and ECM-related genes is in line with the study of Poon *et al*., who showed that knockdown of Rbm24 in HL-1 cardiomyocytes decreases the expression of several Tgfβ- and ECM-related genes, such as Timp1, Ctgf, and Tgfβ2^8^. Together, these findings suggest that Rbm24 regulates ECM synthesis, but the exact molecular mechanism underlying this function of Rbm24 remains to be determined. It is possible that Rbm24 increases the expression of Tgfβ-genes, such as TgfβR1 and TgfβR2 directly, for example by binding to and stabilizing its mRNA transcript, and thereby enhancing Tgfβ signaling, or by regulating the expression of an upstream regulator of Tgfβ signaling. It could also be that Rbm24 acts in its function as a splicing regulator, and regulates the expression of specific isoforms of profibrotic proteins, or it may lead to nonsense mediated decay of spliced targets. Finally, since Rbm24 overexpression is limited to cardiomyocytes, it is also possible that Rbm24 overexpression induces a paracrine factor that, in turn, activates cardiac fibroblasts and collagen synthesis. Many factors secreted from cardiomyocytes, such as Tgfβ, IL-1β, or CTGF^25^, have been described to activate cardiac fibroblast, but it remains to be determined if that is the case here. Future studies are important in addressing how Rbm24 regulates the activation of cardiac fibroblasts and the expression of these ECM genes.

Although fibroblasts have long been considered the main effector cells for fibrosis in the heart, it is increasingly clear that cardiomyocyte Tgfβ signaling also contributes to the fibrotic response^24^. As AAV9 preferentially infects cardiomyocytes in the heart, it seems that Rbm24 overexpression in our study causes cardiac fibrosis by affecting the Tgfβ pathway in cardiomyocytes. There is emerging evidence that, apart from canonical Tgfβ signaling, other pathways contribute to the (cardiac) fibrotic response as well. Small *et al*. have shown that Tgfβ also induces Myocardin-related transcription factor A (MRTF-A) expression in cardiac fibroblasts, which in turn mediates myofibroblast activation and fibrosis, in part by directly activating Col1a2 via a CArG element in its promoter^26^. Yet another pathway that is sufficient to induce myofibroblast activation is the TRPC6-dependent calcineurin pathway^27^. Tgfβ and angiotensin II induce TRPC6 expression via a non-canonical mitogen-activated protein kinase (MAPK) pathway involving serum response factor (SRF). Increased Ca^2+^ influx through TRPC6 then activates calcineurin and NFAT, which promote a myofibroblast activation gene program. Overall, increasing evidence suggests the involvement of multiple pathways in the fibrotic response and it will be interesting for future studies to identify the specific pathways that are affected by Rbm24.

Another set of genes we found to be regulated after Rbm24 overexpression are immune response genes (see Fig. 3B). Also with respect to these immune response genes, there is overlap with the genes that are regulated after knockdown of Rbm24 in HL-1 cardiomyocytes^8^. Several TNFα-related genes as well as several integrins are regulated both after Rbm24 overexpression and Rbm24 knockdown. Since it is known that cardiac fibroblasts can also be activated through increased expression of inflammatory cytokines^28^, it is possible that Rbm24 regulates the fibrotic response indirectly, by increasing expression of these cytokines.

Rbm24 is not the first RBP to be involved in regulating the fibrotic response. It has recently been shown that the RBP Muscleblind-like 1 (MBNL1) promotes myofibroblast differentiation and fibrosis^29^. MBNL-1 is normally very lowly expressed in cardiac fibroblasts, but its expression increases after MI or profibrotic agonists^29^. MBNL-1 then directs myofibroblast differentiation and the fibrotic response through regulation of a network of differentiation and ECM-related genes. The fact that MBNL1 plays such a pivotal role in the fibrotic response opened the door to investigate other RBPs in this entirely new regulatory mechanism of cardiac fibrosis.

It would be of great interest to study the effect of decreased Rbm24 expression, for example in a conditional Rbm24 null mouse. We hypothesize that loss of Rbm24 in the adult heart could attenuate the fibrotic response, for example after pressure overload-induced cardiac remodeling. However, since the Rbm24 null mouse is embryonically lethal^5^, a conditional knockout model to circumvent the embryonic lethality is necessary to examine loss of Rbm24 in a postnatal or adult heart.

AAV vectors have been used extensively for gene delivery in the last decades, and hold promise as a vehicle for human gene therapy^30^. In mice, specifically AAV serotype 9 is suitable for infection of the heart^16,17^, and has, for example, been used to deliver factors to the heart to reduce infarct size after MI^31^, rescue age-related cardiomyopathy^32^, or protect against viral myocarditis^33^.

However, it must be noted that overexpression studies have their limitations, and in that sense also the results from this study need to be interpreted with care. In this regard, it is for example known that high levels of exogenously expressed proteins can lead to non-specific and cardiotoxic effects^34^. In our case, we found a ~15-fold increase in total Rbm24 mRNA levels, while only a subset of cardiomyocytes were infected. This could potentially lead to an 80 to 100-fold increase in Rbm24 mRNA in infected cardiomyocytes. It must be noted, though, that we used immunohistochemistry to calculate the percentage of infected cardiomyocytes. Since this technique lacks sensitivity in detecting low protein levels we may easily have easily underestimated the percentage of infected cardiomyocytes.

Despite concerns of non-specific effects of protein overexpression, we did find an overlap in regulated genes in our study and a previous report describing knockdown of Rbm24 in HL-1 cells^8^. This strongly suggests that at least part of the regulated genes are specific to Rbm24 overexpression. However, to ascertain that increased expression of Rbm24 is causal for the gene regulation and fibrotic effects we see, these data should be complemented with *in vivo* loss-of-function models and molecular analysis of potential targets.

In conclusion, we show that AAV9-mediated overexpression of Rbm24 in the mouse heart increases the expression of Tgfβ- and ECM-related genes and induces cardiac fibrosis. Whether this is through direct regulation of Tgfβ signaling, through cardiac fibroblast activation via increasing inflammatory cytokines, through yet another molecular mechanism, or a combination of these processes remains to be determined.

## Electronic supplementary material

Supplementary Figures

Supplementary File 1
