# Supplementary material for: AAV9-mediated Rbm24 overexpression induces fibrosis in the mouse heart
Source: Sci Rep. 2018 Aug 3;8:11696. doi: 10.1038/s41598-018-29552-x (PMC6076270; doi:10.1038/s41598-018-29552-x)
Supplement: Supplementary file 1 — Supplementary Figures [file 41598_2018_29552_MOESM1_ESM.pdf]

SUPPLEMENTARY INFORMATION

AAV9-mediated Rbm24 overexpression induces fibrosis in the mouse heart

Maarten M.G. van den Hoogenhof[1], Ingeborg van der Made[1], Nina E. de Groot[1], Amin Damanafshan[1], Shirley C.M. van Amersfoorth[1], Lorena Zentilin[2], Mauro Giacca[2], Yigal M. Pinto[1], Esther E. Creemers[1]*

[1]Department of Experimental Cardiology, Academic Medical Center (AMC), Amsterdam, The Netherlands

[2]International Centre for Genetic Engineering and Biotechnology, Trieste, Italy.

***Corresponding author:**
Esther E. Creemers, PhD
Experimental Cardiology
Room K2-104-2
Academic Medical Center
Meibergdreef 15
1105AZ Amsterdam
The Netherlands
+31-20-5663262
e.e.creemers@amc.uva.nl

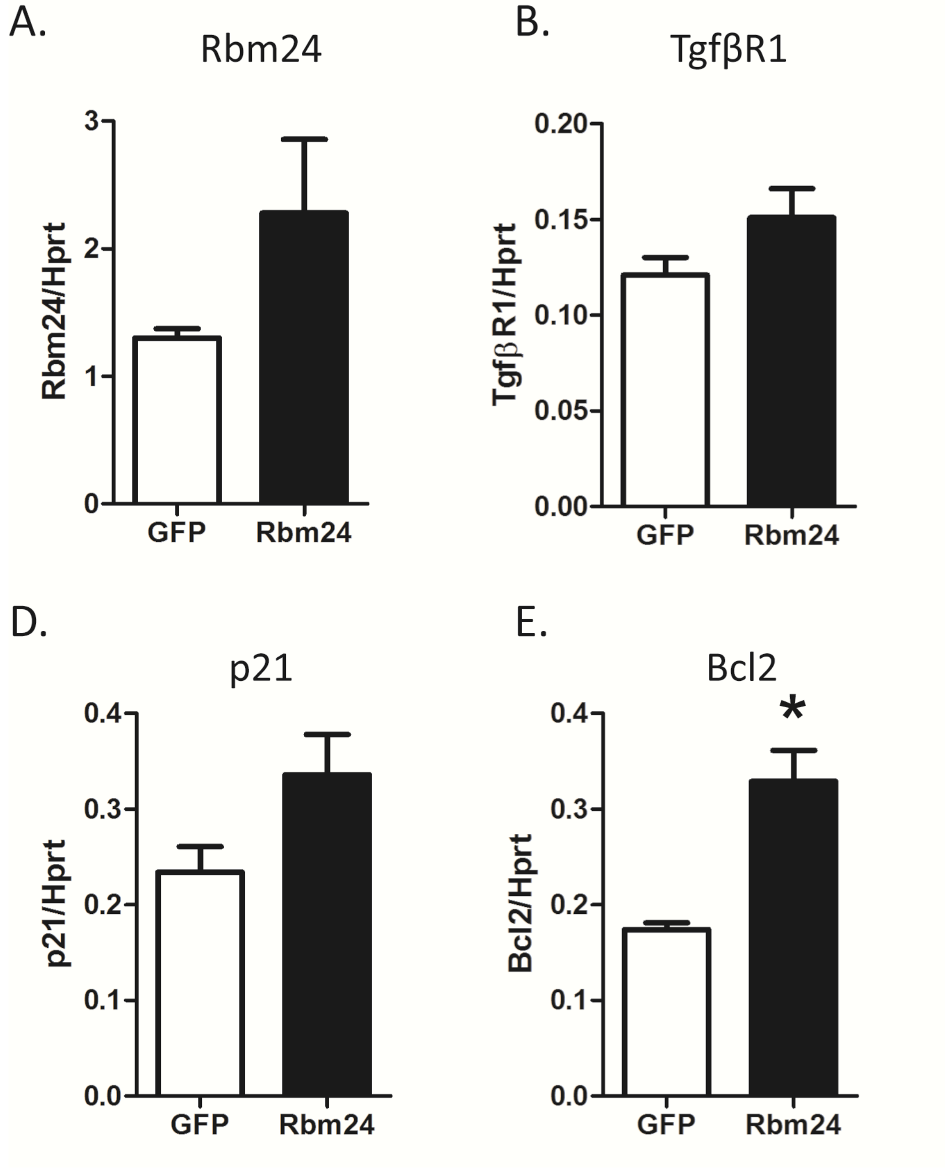

Supplemental Figure 1. qPCR analysis 8 weeks after low dose AAV9 injection. A-D. qPCR analysis in hearts of mice injected with low dose (2 x 10^{12} vg) of AAV9-GFP or AAV9-Rbm24, 8 weeks after injection.

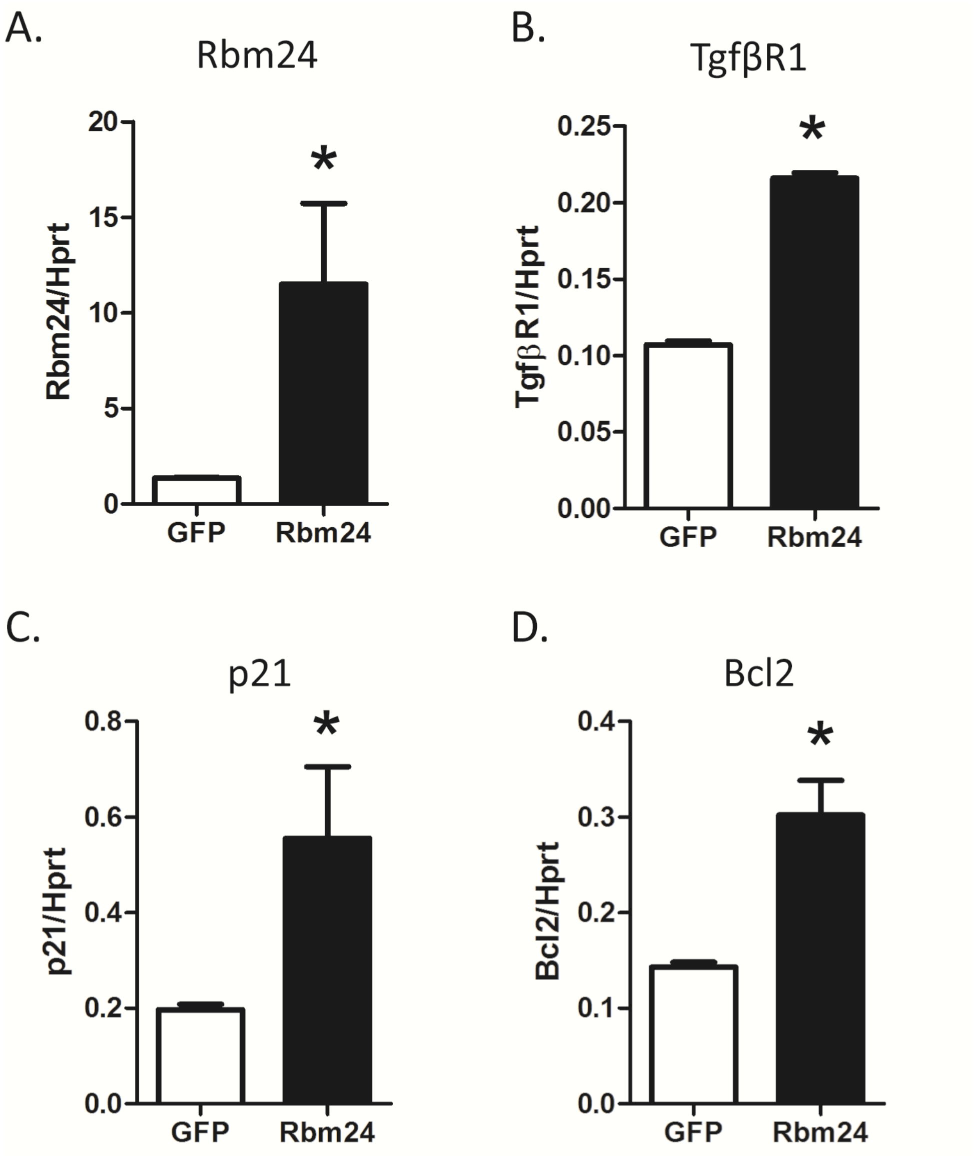

Supplemental Figure 2. qPCR analysis 4 weeks after high dose AAV9 injection. A-D. qPCR analysis in hearts of mice injected with high dose (4×10^{12} vg) of AAV9-GFP or AAV9-Rbm24, 4 weeks after injection.

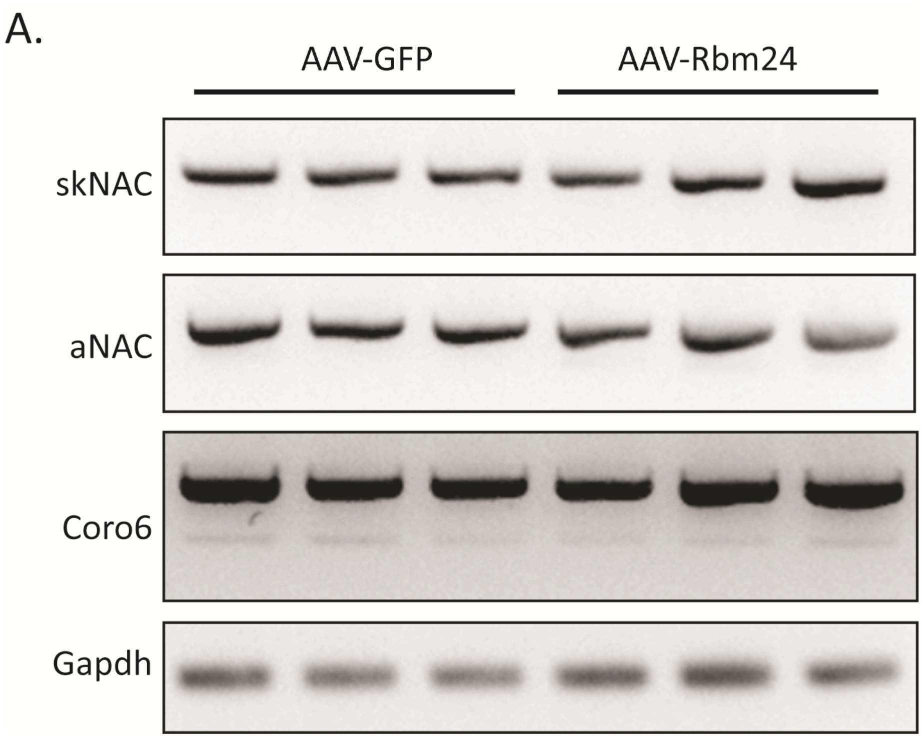

Supplemental Figure 3. Embryonic Rbm24 splicing targets are not altered in AAV9-Rbm24 hearts. RT-PCR analysis of skNAC, αNAC, and Coro6 in hearts of mice injected with AAV9-GFP or AAV9-Rbm24.

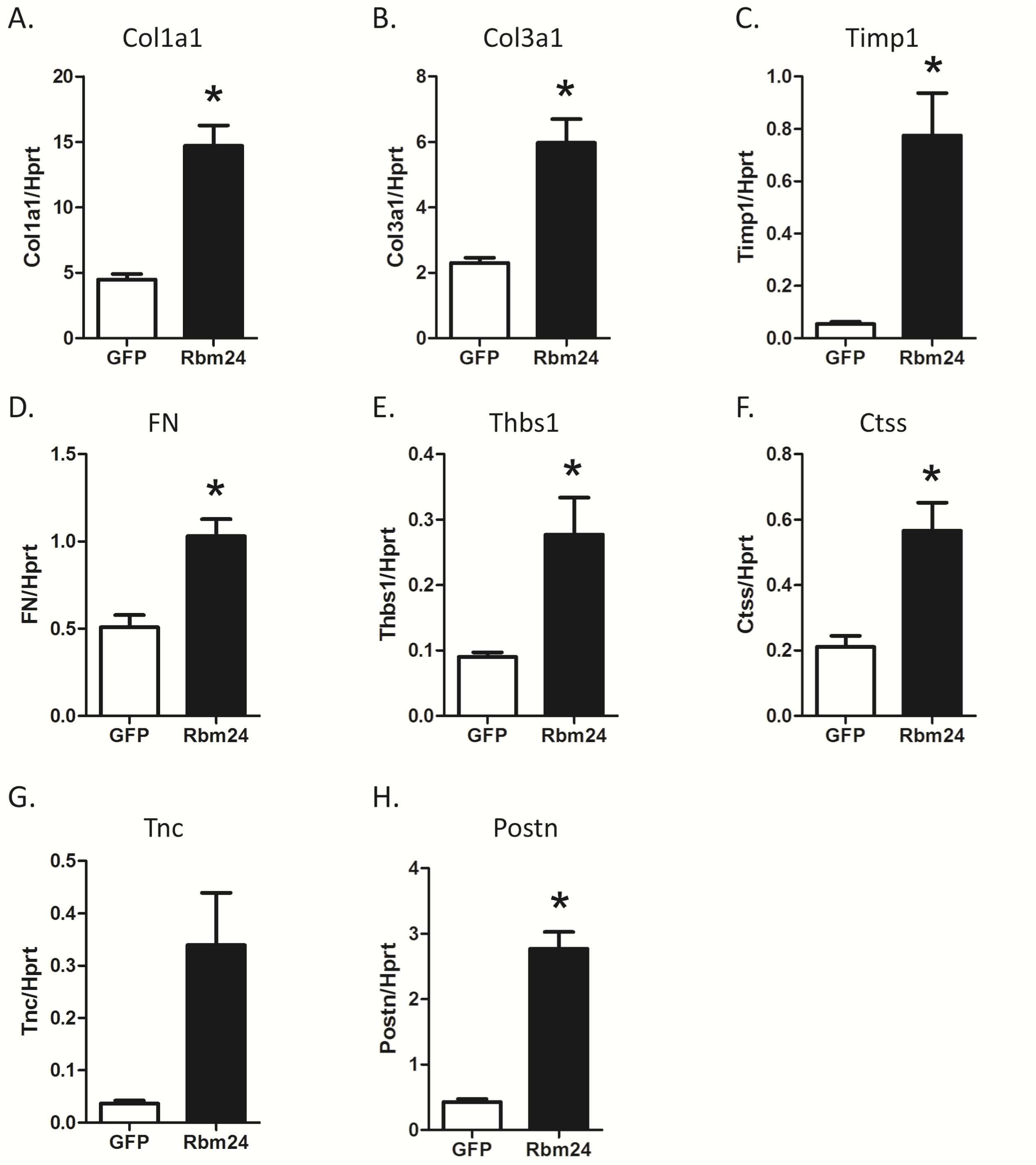

Supplemental Figure 4. Expression of ECM genes 8 weeks after low dose AAV9-Rbm24. A-H. qPCR analysis in hearts of mice injected with low dose (2 x 10^{12} vg) of AAV9-GFP or AAV9-Rbm24, 8 weeks after injection.

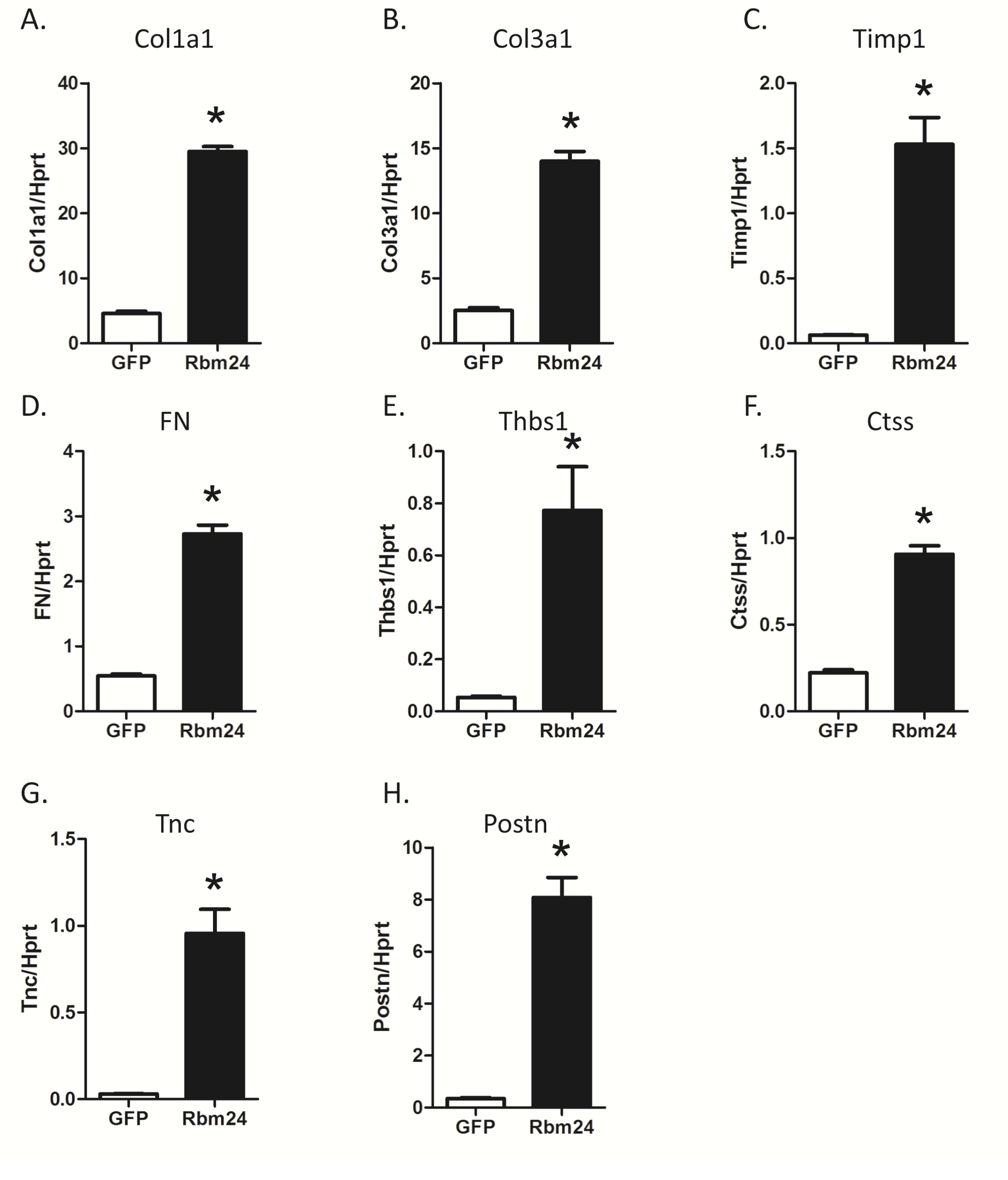

Supplemental Figure 5. Expression of ECM genes 4 weeks after high dose AAV9-Rbm24. A-H. qPCR analysis in hearts of mice injected with high dose (4 x 10^{12} vg) of AAV9-GFP or AAV9-Rbm24, 4 weeks after injection.

Supplemental Figure 6. Uncut gels/blots.

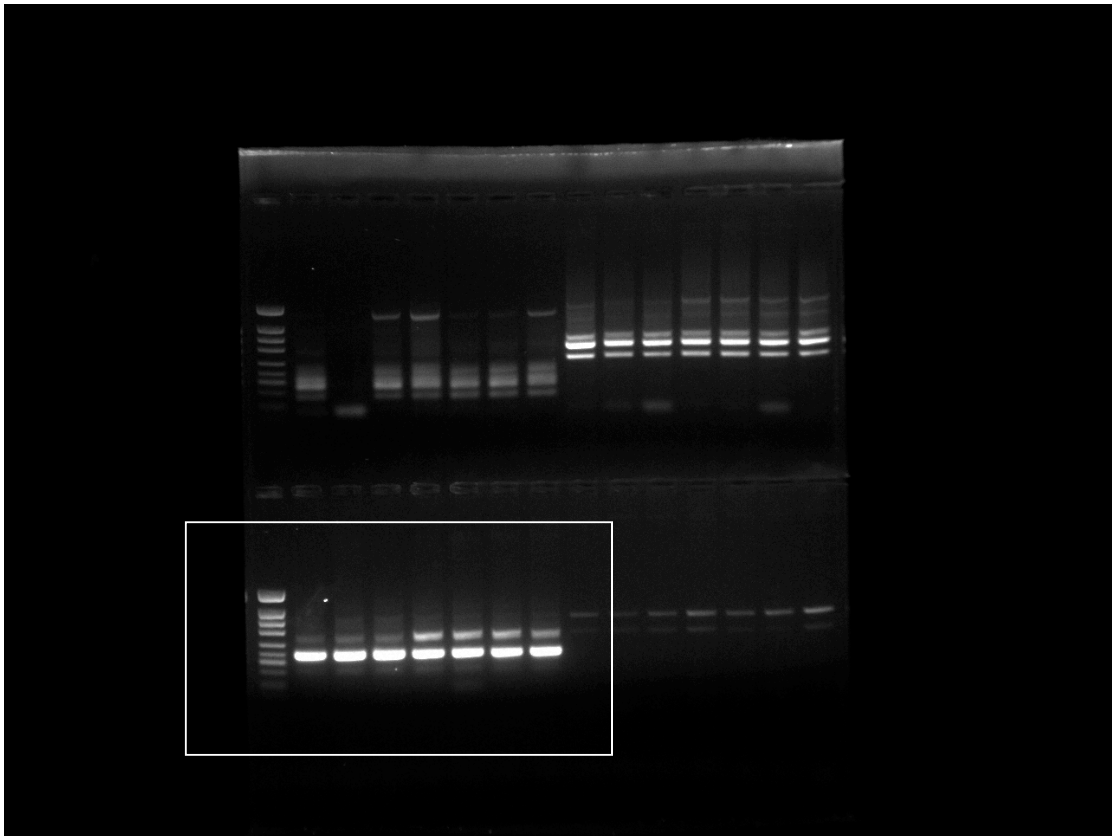

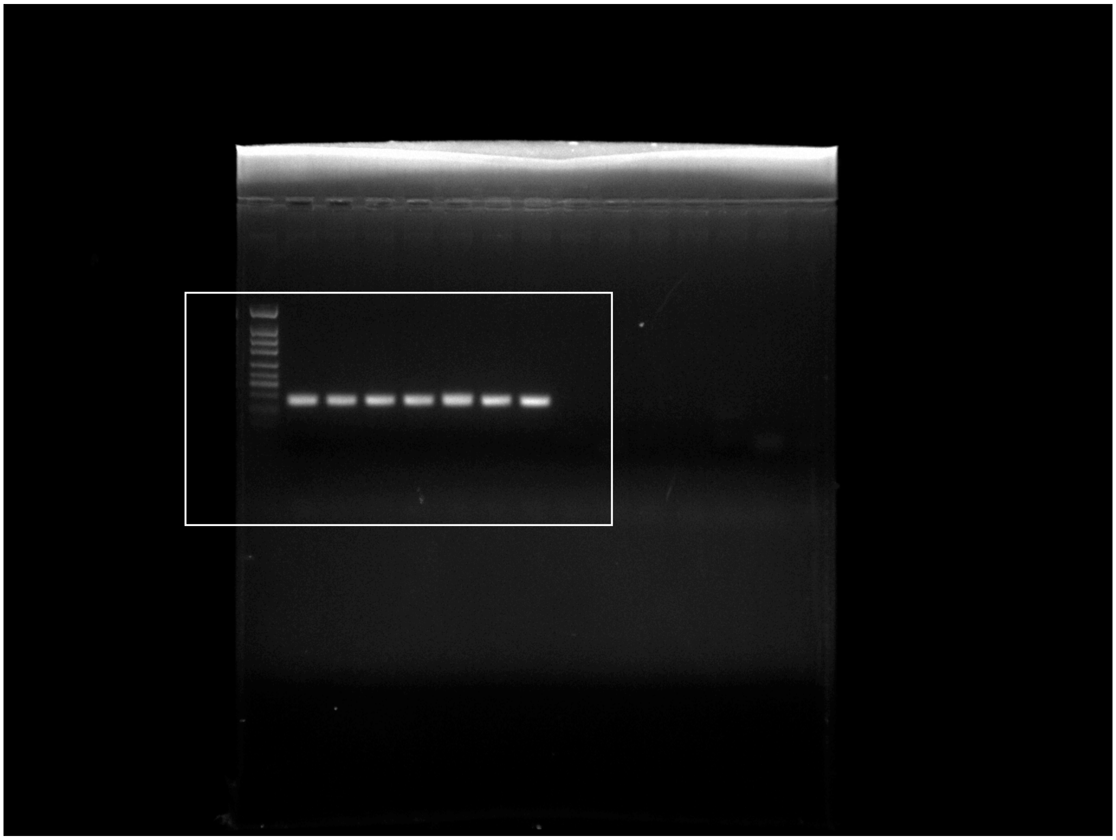

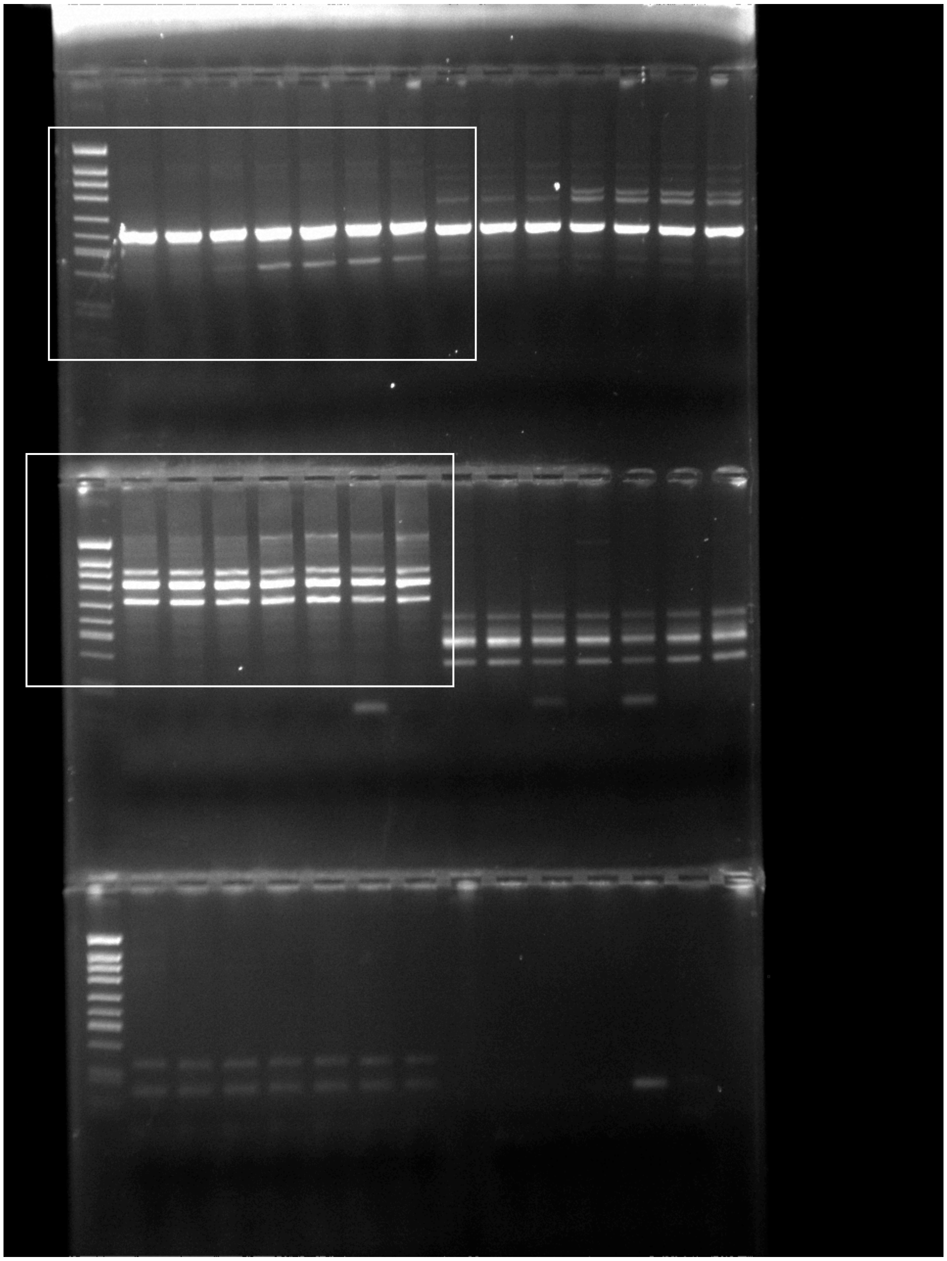

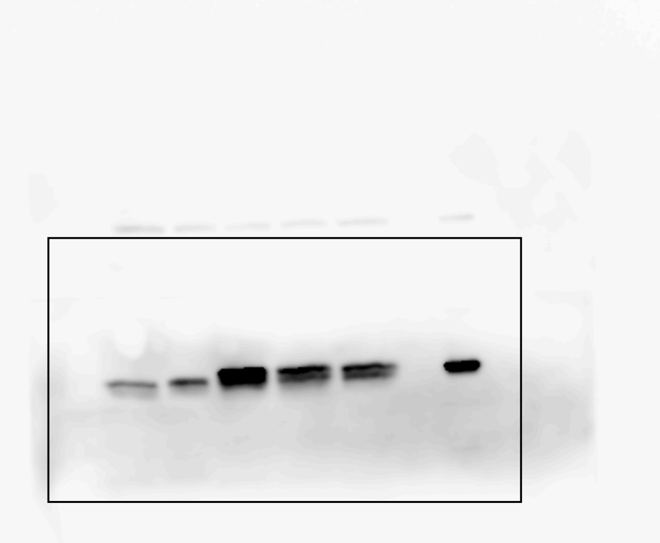

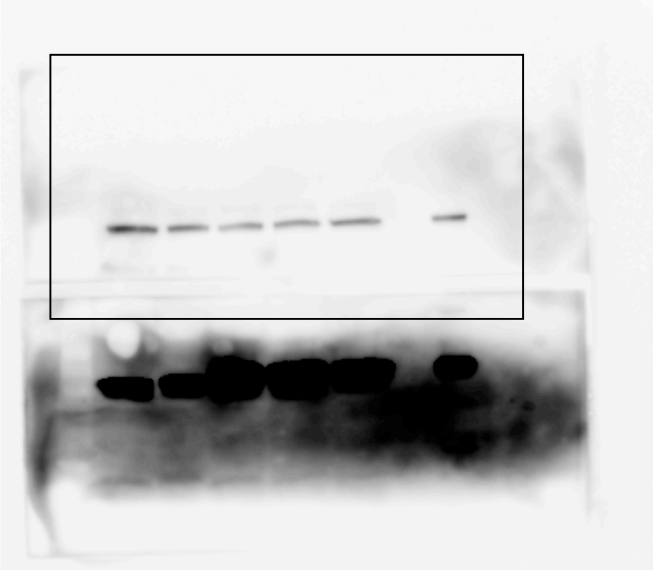

Supplemental Table 1. Primer sequences and antibodies.

**Primers**			
**Gene**	**Forward**	**Reverse**	**Purpose**
Rbm24	GCCAGCCTGCGCAAGTACTTT	GTTGGGATCCTTGCAGGCCCTT	qPCR
Anf	ATTGACAGGATTGGAGCCCAGAGT	TGACACACCACAAGGGCTTAGGAT	qPCR
Col1a1	CTTCACCTACAGCACCCTTGTG	CTTGGTGGTTTTGTATTCGATGAC	qPCR
Col3a1	TCAAGGCTGAAGGAAACAGCA	GATGGGTAGTCTCATTGCC	qPCR
Thbs1	AAGCCCTGTGAAGGTGAAGC	CAGGTGACAGAGCAGATGTC	qPCR
Postn	GGTGATCCCGACTTCAGG	GTTATTTCAACAGGAACTCC	qPCR
Ctss	GGACTACCATTGGGATCTCTGG	CCACTTGGTAGGTATGCATTCC	qPCR
Fn	CACCTACAACCAGTATACACAG	GAGAATCGTCTCTGTCAGC	qPCR
Tnc	CCTGTCCCAATGACTGCAGC	CCTCTGTTACTTCTGTCAC	qPCR
Timp1	CGAGACCACCTTATACCAGC	GGGACTTGTGGGCATATCC	qPCR
p21	AAAGTTCCACCGTTCTCGGG	TCCAGACATTCAGAGCCACAG	qPCR
TgfβR1	AATGGGCTTAGTGTTCTGGG	ACCGATGGATCAGAAGGTAC	qPCR
TgfβR2	AAGCAGACGGATGTCTACTCC	TCCCGCACCTTGGAACCAAATG	qPCR
Bcl2	GCTCTGTGGATGACTGAGTA	CACTTGTGGCCCAGGTATGC	qPCR
Smad5	ACCACTATAAGAGAGTGGAGAG	AACCAGAAGGCTGTGTTGTG	qPCR
Hprt	CCTAAGATGAGCGCAAGTTGAA	CCACAGGACTAGAACACCTGCTAA	qPCR/RT-PCR
Pln	GCTCTGCACTGTGACGATC	TGGAGGCTCTCCTGATAGC	RT-PCR
Pdlim5	GCTGCAGCCAAGAGTGAGC	CGTGTTGCGCTCCACAATGTG	RT-PCR
Ttn	GTCCACGAGGAATGGGAGGA	TTGTCACAGGAACAGGAATC	RT-PCR
skNAC	TACAGAGCAGGAGTTGCCAC	GCAGTTTCAGCTGTTATGGG	RT-PCR
aNAC	TACAGAGCAGGAGTTGCCAC	CTAACTGTGCTTGCTGAGAC	RT-PCR

Coro6	TCATCATCTGGAATGTGGGC	GTACCGAATGCTACTGTCAC	RT-PCR
Gapdh	GGTGGACCTCATGGCCTACA	CTCTCTTGCTCAGTGTCCTTGCT	RT-PCR
**Antibodies**			
Rbm24	SAB2104677	Sigma	WB (1:500)
Flag	F1804	Sigma	IHC (1:1000)
Postn	SC-67233	Santa Cruz	IHC (1:100)
α-actn	A7811	Sigma	IHC (1:200)
α-actn	2310-1	Epitomics	IHC (1:200)
Calnexin	208880	Calbiochem	WB (1:1000)

Supplemental File 1. Gene expression and alternative splicing analysis. See excel File.
